# Current Applications of Absolute Bacterial Quantification in Microbiome Studies and Decision-Making Regarding Different Biological Questions

**DOI:** 10.3390/microorganisms9091797

**Published:** 2021-08-24

**Authors:** Xiaofan Wang, Samantha Howe, Feilong Deng, Jiangchao Zhao

**Affiliations:** 1 Department of Animal Science, Division of Agriculture, University of Arkansas, Fayetteville, AR 72701, USA; xxw033@uark.edu (X.W.); smhowe@uark.edu (S.H.); fdeng@uark.edu (F.D.); 2 Guangdong Provincial Key Laboratory of Animal Molecular Design and Precise Breeding, College of Life Science and Engineering, Foshan University, Foshan 528225, China

**Keywords:** absolute count, high throughput sequencing, flow cytometer, 16S rRNA, qPCR

## Abstract

High throughput sequencing has emerged as one of the most important techniques for characterizing microbial dynamics and revealing bacteria and host interactions. However, data interpretation using this technique is mainly based on relative abundance and ignores total bacteria load. In certain cases, absolute abundance is more important than compositional relative data, and interpretation of microbiota data based solely on relative abundance can be misleading. The available approaches for absolute quantification are highly diverse and challenging, especially for quantification in differing biological situations, such as distinguishing between live and dead cells, quantification of specific taxa, enumeration of low biomass samples, large sample size feasibility, and the detection of various other cellular features. In this review, we first illustrate the importance of integrating absolute abundance into microbiome data interpretation. Second, we briefly discuss the most widely used cell-based and molecular-based bacterial load quantification methods, including fluorescence spectroscopy, flow cytometry, 16S qPCR, 16S qRT-PCR, ddPCR, and reference spike-in. Last, we present a specific decision-making scheme for absolute quantification methods based on different biological questions and some of the latest quantitative methods and procedure modifications.

## 1. Introduction

Subtle alterations in the host environment may induce significant shifts in microbial communities and the corresponding interplays between the host and microbiota [1,2,3,4]. The changes in abundances of certain taxa inhabiting the gut, skin, respiratory system, blood vessels, and other organs can play beneficial roles in human health or cause serious disease [5,6]. Hence, assessing community changes can provide an understanding of the underlying metabolic mechanisms and help direct the development of disease preventatives and therapies. High throughput sequencing is a robust tool used for large-scale profiling of microbial communities to reveal community structural differences. However, most data analyses resulting from the use of this technique are based on relative quantification, with the absolute bacterial abundance being discounted. Inappropriate interpretation from relative quantification may completely change the results of some studies [7]. For example, when two types of bacteria start with the same initial cell number, a treatment that doubles the cell number of bacteria A (while bacteria B remains unaffected) results in the same relative abundance between bacteria A and B (67% and 33%) as a treatment that halves bacteria B (while bacteria A remains unaffected). However, the two treatment effects are completely different [8]. In addition, compositional data using relative abundance is not appropriate for addressing certain biological problems, such as community interactions. Thus, precise quantification of bacterial loads is necessary. Currently available approaches for the absolute count of different specimen types are highly diverse and possess different strengths and weaknesses (Table 1). In this review, we first discuss the concerns and limitations of using relative abundances under different biological conditions. Second, we briefly describe several recent cell- and molecular-based absolute bacterial quantification techniques. Lastly, we provide decision-making directions for method selection regarding different biological questions and challenges for future microbial studies.

## 2. Importance of Absolute Quantification for Biological Questions

Although relative abundance measurement has been widely applied to study community shifts for many biological questions, the absolute bacterial load is crucial for many investigations [9,10]. For instance, bacteria load variations have been frequently reported in both cross-sectional and longitudinal human microbiome studies. Healthy adult human fecal samples have up to tenfold variation (10^10–11^ cells/g) with a 3.8 × 10^10^ cells/g daily fluctuation [11]. In addition, the overall mucosal bacterial loads in patients with gut dysbiosis-related diseases such as Crohn’s disease and inflammatory bowel disease are higher than those in healthy controls [12].

Changes in bacterial density, rather than compositional changes, have been discussed regarding hatching failure in birds. Eggshell-colonized pathogenic bacteria can multiply rapidly on eggshells in a mild environment and subsequently may have the opportunity to infect the embryo through eggshell pores [13]. In some cases, this eggshell infection is bacterial dose-dependent, and low bacterial amounts on eggshells (location-related), even comprising infertility-causing *Neisseria* species, may not cause embryo mortality [14]. However, other similar studies indicated a negative correlation between bacterial densities and hatching success but only in limited locations and avian species [9,15]. Furthermore, eggshell bacterial loads are influenced by various physiological and environmental factors, such as the size of uropygial glands and nest conditions, respectively. Hence, the hatching success rate can also be improved by the modification of these environmental factors, which is indicated by the eggshell total bacterial load. Merely focusing on community structures based on relative abundances will result in overlooking these important biological findings [16].

Microbial community changes in between soil location and development are better revealed using absolute count. Microbial abundances in 110 different soil types collected in the United States showed a greater variation: 30 fold when using phospholipid fatty acid (PLFA) metric and 210 fold when using 16S rRNA gene abundances [2]. In addition, Yang and his colleagues (2018) evaluated the microbial population dynamics in two soil types, a horizontal surface layer soil (Soil) and its parent material (PM) soil. The total bacteria count in the developed surface layer soil was 4.78 times less than the PM soil (3.55 × 10^8^ and 1.7 × 10^9^ cells/g, respectively). When evaluating the individual phyla abundances in these two soils, 20 out of 25 total phyla, including *Acidobacteria* and *Chloroflexi* phyla, showed significant changes from absolute quantification. While only 12 phyla, excluding the two phyla mentioned above, were detected using the relative quantification method. Moreover, at the genus level, 33.87% of the total genera showed opposite changes, described as decreased relative abundance but increased absolute abundance. This was due to the failure to detect the increase in total bacteria count. A similar scenario was observed in the decreased total count. In the same study, sodium azide-treated soil decreased the total indigenous bacteria from 3.85 × 10^8^ to 9.56 × 10^7^ cells/g. Among the 17 classified phyla in the soil sample, 15 phyla dropped significantly based on absolute quantification, while only nine phyla were detected using relative quantification. At the genus level, 40.58% of the total genera exhibited an upregulation trend using the relative quantification method, but downregulation was observed via the absolute quantification method. Hence, data interpretation initiated from relative abundance usually leads to false-positive results, and it is more likely that the change in absolute count of individual members drives the proportion changes within the group [17].

Absolute quantification is more suitable for analyzing complex bacterial interactions in a community. Parasitism, coexistence, predation, mutualism, competition, symbiosis, and antagonism are important inherent interactions that are encountered when exploring inner-microbial correlations [18]. Using traditional correlation methods like Pearson and Spearman when analyzing compositional data is inappropriate [19,20] because most microbiome data starts with a between-group normalization on the observed reads, which forces the data to fall into a compositional fashion and no longer reflects real biological features [21,22]. Microbial loads are not necessarily related to sequencing depth, and a zero in the relative abundance does not necessarily indicate an absence but could also be due to insufficient sampling depth [11,23]. Specific pairwise correlations on bacterial interactions between quantitative microbial profiling (QMP) and relative microbial profiling (RMP) have been studied in Vandeputte et al., 2017. Among 66 fecal samples from healthy volunteers, bacteria loads ranged from 5 × 10^10^ to 3 × 10^11^ cells/g. This 10-fold variation in bacterial density generated significant differences in microbiota interactions using QMP and RMP methods. The QMP network detected 76 significant co-varying genus pairs, including one negative correlation pair (*Phascolarctobacterium-Dialister*, Spearman’s ρ = −0.57), while the RMP network only disclosed ten pairwise correlations, and half of them were negative relationships. Another five positive-correlated genus pairs, *Methanobrevibacter-Akkermansia*, *Bacteroides-Bilophila*, *Alistipes-Barnesiella, Ruminococcus 2-Dorea*, and *Alistipes-Odoribacter,* were detected by both QMP and RMP networks. In the RMP correlation analysis, *Prevotella* had antagonistic effects on *Bilophila*, *Barnesiella*, *Alistipes*, and *Bacteroides*, but these were not validated when the total bacterial count was included in the analysis. Hence, interactive correlations evaluated based on relative and absolute abundance partially coincide, and quantitative taxon profiling has greater biological meaning than compositional taxon profiling [11].

Another challenge posed by relative abundance quantification is microbiome analysis on low-biomass specimens such as the gut microbiota in newborn piglets [24], environmental air microbiotas [25], and medical facilities cleaning [26]. The high sensitivity of high throughput sequencing does not readily distinguish processing contaminants from actual samples. In a single-strain titration study, using Mo Bio PowerMag with a ClearMag bead cleanup step, the target strain was detected as low as five cells per sample. With an initial 50-cell input, only 28.8% of final sequences were aligned to the target strain, while other reads were identified as contamination. When testing samples containing a 500-cell input, the contaminant reads dropped to 10%. Overall, using the specific extraction protocol mentioned above, all samples constantly had 96.9 extraneous contaminant cells [24,27]. The handling of contaminants contributes differently to samples with different absolute cell numbers. Failure to determine absolute cell counts in specimens will reduce the importance of problems caused by contaminants and possibly give rise to false-positive signals.

Hence, the precise and highly sensitive quantification of total bacterial counts or individual taxa show promise for unbiased interpretation of microbial dynamics and interactions. In the next section, we briefly discuss several quantification methods and strategies currently applied in absolute quantification and then provide a specific decision-making scheme based on different biological questions. Also, we describe some of the contemporary optimization techniques used to minimize certain biological obstacles.

## 3. Brief Description of Advanced Absolute Quantification Methods

### 3.1. Fluorescence Spectroscopy

**Figure 1:**
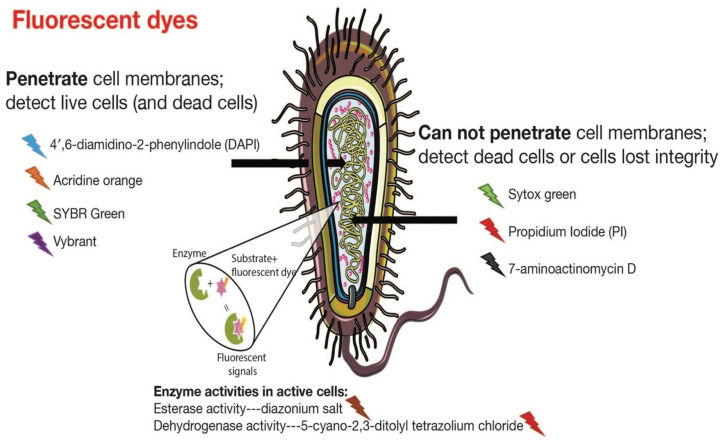
Stains 4′,6-diamidino-2-phenylindole (DAPI; blue color), acridine orange (orange color), SYBR Green I (green color), and Vybrant (red) can penetrate cells and stain the double-stranded DNA. Sytox Green (green color), 7-aminoactinomycin D, and propidium iodide (PI; red color) are cell impermeable and can only stain dead cells or cells that have lost membrane integrity. Active bacteria quantification can be accomplished using certain dyes targeting metabolic functions, such as esterase (diazonium salt) and dehydrogenase activity (5-cyano-2,3-ditolyl tetrazolium chloride).

Dating back to the mid-1700s, bacterial cells were enumerated under a microscope via morphology discernment. Later, there was increasing interest focused on cellular function and metabolic features. As a result, a wide range of fluorescent dyes that bind to different cellular components (nucleic acids, membranes, and proteins) emerged as advanced tools enabling physiological and structural characterizations. Specifically, stains such as 4′,6-diamidino-2-phenylindole (DAPI), acridine orange, and SYBR Green I can penetrate cells and stain double-stranded DNA, thus enhancing the fluorescence of the molecule [28]. The fluorescence strength is related to DNA density and bacterial cell number, which allows the quantification of total bacteria in materials of interest without biological status acquisition. Sytox Green is a high DNA-affinity green dye that cannot penetrate membranes and is thereby used to detect dead cells that have lost membrane integrity [29,30,31]. Other dyes, including 7-aminoactinomycin D and propidium iodide (PI), are also cell impermeable and can only stain dead cells. The application of double nucleic acid stains, such as DAPI (blue fluorescence), allows for simultaneous targeting of both live and dead cells (Figure 1 and Table 1). It is noteworthy that staining nucleic acids for enumerating dead or live cells sometimes has limitations due to complete DNA degradation in dead cells [29]. Certain stains (SYBR, PI, etc.) can bind to both single and double-stranded DNA, as well as RNA but with a lower performance. Other dyes (diazonium salt and tetrazolium salt) that can differentiate active cells by detecting different cellular metabolic activities will be described in a later section.

### 3.2. Flow Cytometry

Flow cytometry, which was referred to as a fluorescence-activated cell sorter in the 1960s, was designed by Bonner, Sweet, Hulett, and Herzenberg. In 1978, the instrument became commercially available and was officially called ‘flow cytometry’. It allowed for the detection of white blood cells through the use of monoclonal antibodies. Within the next couple of decades, the number of applied fluorescent dyes and processing parameters greatly increased [32].

**Figure 2:**
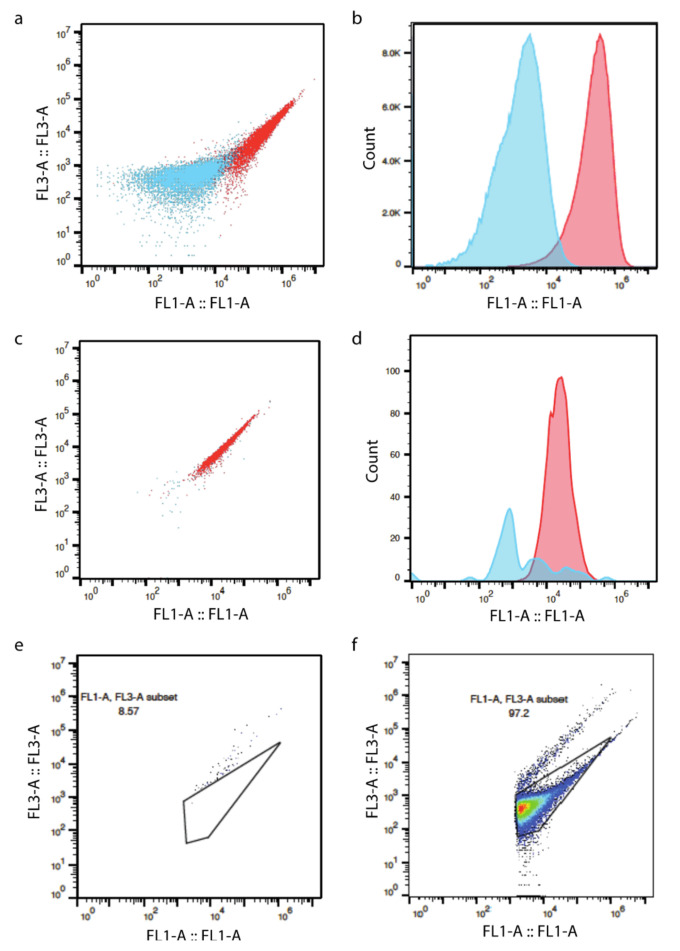
Flow cytometer plots of the same specimens (culture-enriched feces consortium with more background noise: (**a**,**b**); pure culture of *E. coli*: (**c**,**d**)) with or without SYBR green staining (blue: non-stained; red stained). Flow cytometer plotting of buffer was used as gating guidance to exclude noises (negative control, (**e**)). Gating strategy used for bovine respiratory microbial quantification (**f**).

Flow cytometry is a multi-purpose detection device. Developed from fluorescence spectroscopy, it drives cells through a small orifice individually to initiate complex detection of each cell [33]. The narrow flow channel is equipped with a multi-color laser beam that can detect each passing cell. Electronic signals such as emitted fluorescence and forward and side scatter light are collected by a computer and translated into specific cellular information. Forward and side scatter light detect cellular size and surface evenness, whereas fluorescence provides positive signals that differentiate cells from non-relevant particles [34]. This sophisticated setting promises comprehensive detection of multiple physiological characteristics simultaneously with high speed and high resolution, thus qualifying as a widely used bacterial quantification method for heterogeneous microbiota specimens. The most commonly used stain for bacteria is SYBR green (1 or 10 μL/mL; 1:100 diluted in DMSO; 15 min at 37 °C) with the emission detector set at FL1 533/30 nm and FL3 > 670 nm [35]. Forward and side scattered signals are recorded simultaneously to assist in signal identification. Flow speeds from 17 to 200 μL/min were used in different studies [35,36,37]. Background disturbance is a problem because minerals, plant particles, and bacterial debris emit weak fluorescence signals without staining [38]. SYBR green is a strong stain that can greatly increase the fluorescence signal, which helps distinguish bacterial cells from the ‘background’ (Figure 2a–d). Vandeputte et al., 2017 set a threshold of 2000 on the FL1 channel to exclude non-significant background signals, also known as the negative signal. Under some circumstances (e.g., bovine respiratory microbiome quantification), a threshold on the FL1 channel is not enough to rule out all irrelevant compounds contained in the buffer. Hence, running a negative control of only buffer is necessary to guide the gating strategies (Figure 2e,f). Furthermore, the flow cytometer-based bacteria enumeration method has a low detection limit between 10^3^–10^4^ CFU/mL, which allows the detection of a wide range of sample types, such as fecal, soil, and aquatic organisms [11,39] (Table 1).

### 3.3. Spike-In with Reference Markers

Another fast and advanced method often combined with high throughput sequencing is spiking the specimens with internal microorganism markers (Table 1). The cell numbers and target nucleotide sequence of the internal markers are known, which allows for the calculation of the absolute bacterial number. This strategy simulates the capture-mark-recapture method used to evaluate wild animal populations, in which each processed sample spiked with a known amount of a microbial marker. The original bacterial load is calculated by dividing the absolute internal markers count by the relative abundance of internal markers [40]. There are different types of spiking markers that were used previously, including indigenous microorganism, synthetic, and heterogeneous markers. The marker selection and spiking strategy can be challenging since only validated markers can be used to achieve reliable results. More details of marker use will be discussed in later sections.

### 3.4. 16S qPCR and qRT-PCR Quantification

Polymerase chain reaction (PCR), invented in the 1980s, was a major breakthrough and is used across a wide range of fields in biological science. The technique amplifies specific nucleic acid sequences in the template by millions-fold, which can be detected by incorporating certain fluorescent labels [41]. The target template can be genomic DNA or complementary DNA (reverse transcription product of mRNA, cDNA). The PCR technique subsequently evolved to possess more robust capabilities such as the enumeration of nucleic acid sequence copies such as quantitative polymerase chain reaction (qPCR).

The 16S rRNA qPCR, a frequently used tool for gene expression detection and quantification in real-time, is commonly used for both absolute and relative quantification [42,43]. Absolute quantification is accomplished using a standard curve produced by amplifying a series of diluted DNA standards of known concentrations and copy numbers to convert threshold cycles to the absolute quantity of the target gene (16S rRNA gene). Circular plasmid DNA containing the gene of interest [42,44] and reference organism DNA [45] at different serial dilutions have been widely used as DNA templates to generate standard curves. 16S qPCR has been widely used as a complementary absolute quantification method that can be integrated into next-generation sequencing but provides only relative quantification. In parallel with normal library construction targeting the 16S rRNA hypervariable regions, the same universal bacterial primer set for next-generation sequencing was used in qPCR for total bacterial load quantification in each sample. Of note, when reference organism DNA is used as the standard, the total abundance for each taxon should be corrected for differences in the 16S rRNA copy number, which can be found in the rrnDB database [45]. Reverse transcription qPCR (qRT-PCR), which targets mRNA in actively metabolizing cells, can also be used for absolute quantification with a higher sensitivity compared to regular qPCR. Here, mRNA is converted to cDNA via reverse transcription and subsequently quantified using qPCR [46]. qRT-PCR has been used for bacterial detection and quantification when it is necessary to determine the number of active bacteria, such as in clinical settings, food industry, and activated sludge (wastewater remediation) [47,48,49,50]. Using 16S rRNA qPCR and qRT-PCR for absolute quantification is simple, economical, and highly sensitive [51].

### 3.5. Droplet Digital PCR (ddPCR)

**Figure 3:**
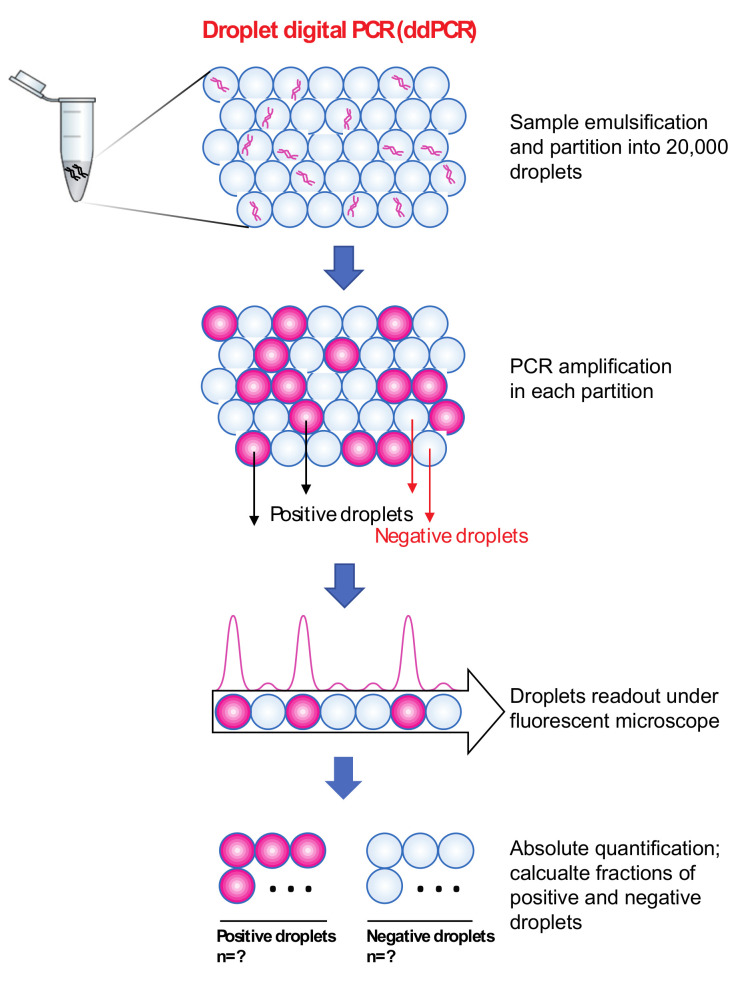
General workflow of droplet digital PCR. DNA template was individually partitioned into approximately 20,000 ‘water-in-oil droplets’. After PCR amplification, flow cytometry is used to read the droplets. Each droplet is then scored as either 1 or 0, which signifies positive or negative and represents this method’s digital facet. These values are then used for absolute quantification using Poisson statistics.

Another PCR method used for absolute quantification is droplet digital PCR (ddPCR; Table 1), which was created in 2011. In this method, each 20 µL PCR reaction is distributed into approximately 20,000 ‘water-in-oil droplets’, which allows for the amplification of a single template. After amplification, flow cytometry is used to read the droplets. Each droplet is then scored as either 0 or 1, signifying a negative or positive digital facet of this method. These values are used for absolute quantification, which is calculated using Poisson statistics, and therefore, no standard curve is required [52,53,54,55] (Figure 3). ddPCR can amplify as little as one copy per droplet [56]. Multiple studies have used ddPCR to enumerate total bacteria (using 16S primers) or specific classes of bacteria (using species- or taxon-specific primers). Ziegler et al., 2019 used ddPCR to quantify the bacterial DNA in the blood from patients with bloodstream infections. The results suggested that ddPCR could be used to quantify both the broad range of bacterial load and species-specific ddPCRs (*Staphylococcus aureus*, *Streptococcus pneumoniae*, and *Escherchia coli*) with similar sensitivities [57]. Studies have found that overall qPCR and ddPCR results are comparable, but that ddPCR was able to enumerate samples with lower target concentrations than qPCR when using *Lactobacillus* specific primers [53]. Also, the detection limit was lower and the detection range was wider for 16S ddPCR when compared to 16S qPCR [54]. Furthermore, compared to 16S qRT-PCR, 16S ddPCR was 10x more sensitive [55]. In addition, ddPCR can accurately quantify samples with a low abundance of the target gene and reduce background noise in negative control samples. This is demonstrated by ddPCRs significantly lower coefficients of variation and its ability to detect lower numbers of 16S rRNA gene copies compared to qPCR, while the sample 16S rRNA gene copies were the same [54].

## 4. Decision-Making Regarding Different Biological Questions

### 4.1. Differentiation between Active and Dead Cells

**Figure 4:**
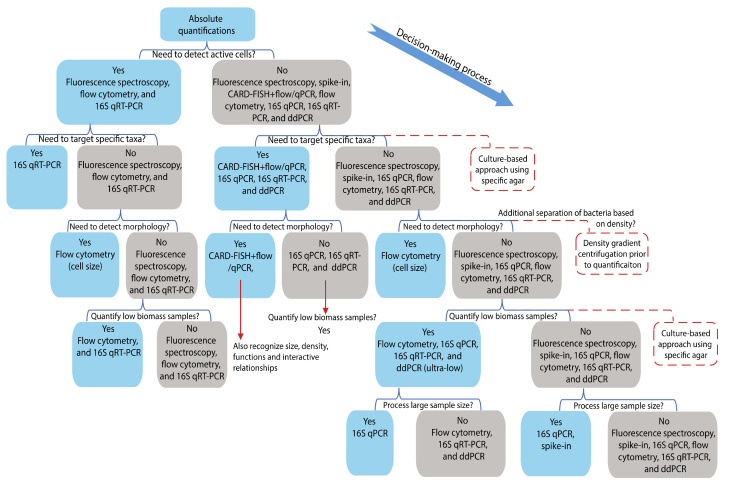
Decision-making process of absolute quantification methods.

Among these quantification methods, fluorescence spectroscopy, flow cytometry, and 16S qRT-PCR possess the ability to distinguish dead or enzymatically active cells from total cells in a sample (Figure 4). Fluorescence spectroscopy and flow cytometry mainly exclude the dead cells with incomplete membranes through the utilization of membrane-impermeable dyes such as Sytox [58]. Roth et al., 1997 enumerated the beta-lactam antibiotic-resistant *E. coli*, contingent on cell membrane integrity, using flow cytometry and Sytox [31]. In addition, active bacterial quantification can be accomplished using certain dyes targeting metabolic activities, such as esterase and dehydrogenase activity [59,60]. Esterase activity is an indirect bacterial quantification method detecting fluorescence produced from cleaved esterase substrates by a local enzyme. Dehydrogenase activity can be measured using histochemical indicators, such as tetrazolium salt (5-cyano-2,3-ditolyl tetrazolium chloride), a typical indicator for respiratory activity that is used for active bacterial quantification [61,62]. However, metabolic activities do not indicate the viability of bacteria, especially when energy-independent enzymatic indicators are used. Certain enzyme activities can continue for more than a week after bacterial deactivation [59]. These differences can lead to discrepancies in data gathered using different metabolism-based quantification methods.

Since transcription only occurs in live cells, qRT-PCR targeting mRNA can be used to quantify viable bacteria. Ma et al., 2018 used qRT-PCR and a standard curve to detect and quantify viable bacteria that have a high tolerance to antibiotic chemotherapy on spacers used in “two-stage revision total knee arthroplasty” [47]. Dolan et al., 2009 used qRT-PCR to quantify total viable bacteria present on beef carcasses to develop an alternative to the total viable count culture technique used to predict shelf life, and no significant differences were found when comparing the two quantification methods. However, they targeted the ribonuclease-P transcript rather than 16S rRNA [48]. Additionally, Bui et al., 2012 targeted 16S rRNA, as well as *dnaJ* and *ciaB* mRNA, utilizing qRT-PCR to detect and quantify *Campylobacter jejuni* in ‘naturally contaminated’ and spiked chicken fecal samples to determine if qRT-PCR could be used as an alternative to *Campylobacter* culture-based quantification. They concluded that qRT-PCR performed on the spiked fecal samples to measure *C. jejuni* survival was similar to results obtained via the culture method [49]. Furthermore, Johnston and Behrens., 2020 used 16S qRT-PCR, 16S qPCR, and next-generation sequencing to analyze the activated sludge microbiome from material used in wastewater remediation that were processed throughout the year [50]. Therefore, qRT-PCR serves as a sound approach to quantify live bacteria cells.

### 4.2. Absolute Quantification of Specific Taxa of Interest

Absolute quantification of specific taxa can be achieved with a simple calculation: multiplying the relative abundance of the taxa generated by 16S rRNA amplicon sequencing with total cell counts. However, various technical biases could potentially be introduced into the results when using different absolute and relative quantification mechanisms, such as 16S rRNA copy number discrepancy and PCR primer coverage and specificity [63]. 

Alternatively, 16S qPCR, 16S RT-qPCR, and ddPCR could all directly detect taxa of interest using specifically designed primers without the need for total bacterial quantification (Figure 4). Gobert et al., 2018 used both ddPCR and qPCR to quantify lactic acid-producing bacteria in piglet feces using *Lactobacillus*-specific primers [53]. Jian et al., 2020 applied an additional qPCR technique on four 10-log-fold diluted reference bacteria (i.e., *Bacteroidetes*, *Clostridium cluster XIVa*, *Bifidobacterium,* and *Escherichia coli*) to quantify their absolute cell counts in feces, which were highly correlated with their absolute quantifications achieved by next-generation sequencing and total cell numbers. The total abundance for each taxon was corrected based on the 16S rRNA copy number, which could be found in the rrnDB database [45]. In addition, Matsuda et al., 2009 analyzed the predominant and subdominant members of the fecal microbiota from 40 healthy individuals using both 16S and 23S qRT-PCR and found that qRT-PCR was more sensitive than qPCR [64]. However, it is important to note that 16S rRNA transcript copy number is more indicative of protein synthesis potential, while 16S rRNA gene copy number is more indicative of the overall sample cell count [50]. Another concern associated with qRT-PCR is the risk of RNA degradation [65].

Another method that can directly enumerate specific taxa is fluorescence in situ hybridization (FISH), which recruits a fluorescently labeled probe that can hybridize the complementary sequences in the targeted cells. Because of its high sensitivity, it has been used to detect and/or quantify low abundance microbes and those with “low ribosome content” [66,67]. Although FISH provides only a relative abundance measurement, it can be combined with flow cytometry and microscopy to determine the absolute abundance of each taxon of interest independently of all bacterial members [63,68,69]. Furthermore, applying FISH with multiple dye-probe combinations allows for the detection of different types of bacteria simultaneously. Kuo et al., 2021 designed four different probes using dyes Cy3, FAM, Texas red, and Cy5, respectively, and successfully detected four coliform bacteria (*E. coli*, *K. pneumoniae*, *E. aerogenes*, and *C. freundii*) in water samples [70]. Detecting rare taxa in a community using FISH requires a great number (500–1000) of counted cells to maximize the precision of the targeted count [67].

### 4.3. Absolute Quantification of Low Biomass Bacterial Samples

Enumeration of low bacterial counts on sterile and non-sterile pharmaceutical equipment and products requires techniques with high sensitivity and precision, as well as the ability to exclude background noise. Quantitative methods that have been widely used in such areas include cell culture, qPCR, ddPCR, and flow cytometry [51,71,72,73,74,75] (Figure 4). Culture-based quantification is impractical due to its low sensitivity, inaccuracy, intense labor, and immense time consumption. PCR-based approaches possess high sensitivity and short turnaround time, while flow cytometry, besides the benefits previously mentioned, can distinguish cell populations based on physiological features. Ahn et al., 2020 further compared these four absolute quantification approaches using twenty serial-diluted (1, 10, 100, and 1000 CFU/mL) strains of *Burkholderia cepacia*. Both qPCR and ddPCR successfully measured low genomic DNA (*B. cepacia* PC783 and *B. cenocepacia* J2315) concentrations at 14.4 target copies/μL. ddPCR had a 10-fold greater sensitivity and was able to detect as low as 1.4 copies of *B. cenocepacia* J2315 DNA [51]. The high sensitivity of ddPCR was also demonstrated by Zeng et al., 2020 who used it to accurately quantify *Streptococcus agalactiae* levels as low as 5 pg/μL [76]. However, Ahn et al., 2020 found that ddPCR had a high detection limit, requiring DNA at 1.4 × 10^5^ target copies/μL [51]. In another study, Sze and colleagues also found that samples with estimated DNA copy numbers greater than 10^5^ must be diluted before quantification [54], which greatly increases sample processing time. Therefore, ddPCR requires a primary concentration estimation prior to dilution factor determination and 16S rRNA copy number conversion after dilution [77]. Overall, flow cytometry, qPCR, and ddPCR had greater detection capabilities (70.3%, 43.5%, and 66.8%, respectively) than culture-based methods (18.3% to 25.5%) when using nuclease-free water as the common suspension medium. 

Culture-enriched molecular profiling serves another important biological role. It can reveal low biomass and rare taxa in a bio-environment that failed to be detected by next-generation sequencing because of shallow sampling depth [78]. Although such methods provide less value in terms of absolute versus relative quantification, they can identify otherwise undetected bacterial types, which may play important roles in diversity and community dynamics.

Enumeration of environmental air microbiotas still depends on culture and microscopy-related approaches in most cases due to the ultra-low biomass of these types of samples [75,79]. Extra steps are needed for microbiota amassment prior to quantification and sequencing to obtain enough DNA for processing, and these steps can influence DNA yields for a variety of reasons. Luhung et al. 2021 evaluated the effects of air flow rate, time interval, sonication wash, detergent, and other adjustments on bacterial recovery and DNA yield during the amassment. Specifically, the filter paper used for collecting air microbiota can be washed with PBS buffer to achieve a better microorganism recovery rate compared to direct DNA extraction from the filter paper. Additional sonication during washing had no impact on quantitative and metagenomic outcomes; however, washing with detergent increased the total DNA yield for both bacterial and fungal counts and their compositional profiling. Furthermore, overnight pre-incubation of samples at 55 °C greatly improved cellular lysis, especially for fungal analysis [25].

### 4.4. Rapid Quantification for a Large Number of Samples

Although ddPCR, flow cytometry, CARD-FISH, and cell culture can fulfill most quantification requirements for various kinds of biological studies (high sensitivity, specific taxa targeting, and low biomass detectability), the sample preparations and quantification procedures are very time consuming and laborious, especially for large sample sizes (Figure 4). In contrast, qPCR, qRT-PCR, and spike-in require fewer steps and shorter operation times. qPCR and qRT-PCR, combined with a standard curve, can be used as complementary quantification methods for total bacterial counts that can be integrated with the downstream compositional analysis generated by sequencing. The spike-in method can be integrated into next-generation sequencing. It can provide absolute and relative quantifications simultaneously with simple operational steps, enabling rapid processing of many samples. However, spiking marker selection and spiking concentration are critical to the sequencing quality and the accuracy and sensitivity of the quantification.

The spiked internal reference(s) should either not be or be rarely present in real specimens. Implementing this will avoid the addition of irrelevant reads to the samples and prevent further changing of the community structure. Background calibration of internal reference-like microbes for all samples largely increases the workload and complexity. Yang and his colleagues applied a spiked marker *Escherichia coli O157:H7* into specimens for Miseq sequencing, which later was designated as “*Escherichia* genus”. This genus was also present in the soil samples; thus, additional steps were needed to distinguish the indigenous *Escherichia* genus from the total detected amount [40]. In another study by Lou, who used the commercial strain *Escherichia coli O157:H7* (EDL933) as an internal reference, a single-copy gene, *fliC,* was used for absolute quantification of EDL933 in a soil specimen using a standard curve of serially diluted *fliC* gene-inserted plasmids [80]. Although the indigenous reference-like bacteria in both the above-mentioned studies turned out to be negligible, extra caution was necessary when using strains that were already present in a specimen. Besides, adding those strains contributed to dose-dependent variations in the overall bacterial community structure, making the situation more complex [40].

Spiking non-relevant bacteria into specimens does not require indigenous calibration once the absolute bacterial load of the internal reference can be specifically and accurately quantified. In previous spike-in studies, *Salinibacter ruber* (hypersaline environments), *Rhizobium radiobacter* (soil), and *Alicyclobacillus* (thermo-acidophilic) were chosen as internal references for quantifying gut microbial specimens [7], while *Aliivibrio fischeri* or *Thermus thermophiles*, mostly found in marine animals, were used in soil specimens [2]. Titration in both studies was achieved by spiking the same number of internal references into serially diluted specimens or spiking serially diluted internal references into the same amount of sample to validate the accuracy of the spike-in quantification method. Overall, there was a linear relationship (0.6 < |r| < 0.96) between estimated total bacteria load (by internal references) and the total sample amount used for DNA extractions. This relationship indicated the internal reference spike-in method was a success, although variations were discovered when using different reference strains. In order to avoid altering the microbial community structure by incorporating internal references, 16S rRNA with artificially replaced variable regions was used as an internal reference [10]. This synthetic internal reference can be detected by high throughput sequencing techniques with real samples because it maintains the 16S rRNA conserved region. The aligned sequence cannot be classified to any database in NCBI, est, and est_human. Also, microbial structures were not affected by the insertion of synthetic internal references indicated by OTU richness and Bray–Curtis dissimilarity analysis.

The amount of internal reference used determines the calibration accuracy and sequencing quality. Overloading internal references will reduce the total reads generated from real microbial samples and decreases the sequencing depth, whereas spiking a low amount of internal references may not serve as a functional internal control. In the 10-fold titration model, adding 1% internal reference increased the coefficient of determination of the linear regression model from 0.79 to 0.91 compared to what was achieved by inserting 0.1% internal reference [2]. Moreover, spiking a serially diluted internal reference (except synthetic references) into soil specimens contributes to various community structure alterations, leading to bias in the original sample [40]. Hence, preliminary testing for a qualified range of internal reference content is important, especially in specimens with a large amount of community variation.

Adding internal references to a sample before (whole cells) or after (extracted DNA) processing can result in a slightly different calibration mechanism. Adding such a reference before DNA extraction can normalize the variations generated from DNA extraction [10]. Moreover, commercial extraction kits and protocols have different efficiencies regarding the recovery of total genomic DNA [27]. Overall, high throughput sequencing with an accompanying universal internal reference is a functional method for determining the absolute bacterial counts in samples. 

### 4.5. Absolute Quantification of Bacteria Based on Other Features 

Under certain circumstances, a subgroup of bacteria with specific molecular features such as morphology, function, and interactive activity may need to be distinguished (Figure 4). For instance, in aquatic environments, bacteria potentially carry either low or high nucleic acid content (LNA and HNA), representing two main groups with different metabolic and ecological properties [81,82]. LNA bacteria have stronger resistance to the stresses encountered in severe marine environments than HNA, while HNA have a greater metabolic rate than LNA, except in barren environments [83]. Studying the distribution of these two bacterial groups could provide more insights into interpreting the relationships between microorganisms and the marine environment. Hence, for such purposes, a single-cell enumeration method using flow cytometry that records physical and chemical characteristics is frequently used for most aquatic microbiome studies such as sea, sewage, lake, and drinking water [37,84,85,86]. Specifically, green fluorescence intensity from HNA is 3–5 times greater than LNA and serves as the main separation criteria for these two cell populations [37,84,87]. The LNA and HNA can be gated by a duo-parameter scatter-plot of green fluorescence (520 nm) against side scatter [37]. Using this gating and staining strategy, the distribution of LNA and HNA was revealed to be significantly associated with the season, salinity, and chlorophyll-α [88,89]. 

Another technique that can be used prior to compositional analysis to separate the substances based on size (rate-zonal centrifugation) and density (isopycnic centrifugation) is density gradient centrifugation. This allows direct downstream analysis of each subpopulation. This method has been used to separate aquatic microbes that differ in cell size and metabolic properties, including isolating viruses of interest from background noises [90,91,92,93,94]. It can be combined with other absolute quantification approaches such as flow cytometry and qPCR to enumerate bacterial groups with similar morphology.

By targeting functional or phylogenetic marker genes, qPCR can enumerate groups of bacteria with certain biological functions. Blaud et al., 2021 quantified ammonia-oxidizing bacteria abundances that play an important role in nitrification by targeting the *amoA* gene via qPCR [95]. In another published study, absolute copy numbers of nitrogen-related functional genes (*amoA*, *narG*, *napA*, *nirS*, *nirK,* and *nosZ*) were quantified by qPCR to evaluate the effects of phosphogypsum and medical stone on nitrogen gas emissions during aerobic composting [96].

Catalyzed reporter deposition (CARD)-FISH is an improved version of FISH that has a 200-fold greater sensitivity than FISH and can be used to analyze physiology, metabolic activity, and ecological interactions [67,68,97,98]. Besides the benefits of specific taxa detection, double CARD-FISH using multiple fluorescent tracers allows the exhibition of the processes of interactive activities directly among communities, such as predator-prey interactions [99]. Using CARD-FISH together with Taq-sequencing allowed the morphological visualization of certain rare or uncultured taxon-specific cells (*Rozellomycota* s./.) [100]. Furthermore, this approach can be combined with flow cytometry and next-generation sequencing to explore compositional dynamics and shed light on ecological interactions between different microbes [68,101].

## 5. Conclusions

In conclusion, compositional profiling based solely on relative abundance that is generated by 16S rRNA sequencing can lead to an incorrect interpretation of microbiota dynamics. Recently, methodologies regarding rapid and precise absolute counts have gained a renewed prominent focus. Fluorescence spectroscopy, flow cytometry, and 16S qRT-PCR possess the capabilities to enumerate only viable bacterial cells. Additionally, a bacterial community can be quantified into subgroups based on morphology, size, density, cellular functions, and interactive relationships. These specific features can be recognized using flow cytometry, qPCR, and CARD-FISH with or without extra sample processing, such as density gradient centrifugation. Depending on different sample types, cell culture, qPCR, ddPCR, and flow cytometry have been utilized for absolute quantification of samples with low biomass. Among these methods, ddPCR had the lowest detection limit. When direct quantification of specific taxa is needed, 16S qPCR, 16S RT-qPCR, and ddPCR can be applied using specifically designed primers. FISH combined with flow cytometry also offers an advanced approach for targeting specific taxa. However, some of these quantification approaches are laborious and increase the required workload, especially when targeting specific taxa. When absolute abundance is preferred, spiking internal references into raw specimens offers a practical and time-saving method for quantification in studies requiring a large number of samples. However, choosing the appropriate spiking reference selection and concentration is crucial to maximizing the sequencing quality, accuracy, and sensitivity of the quantification. We have discussed the most widely used bacterial load quantification methods, including fluorescence spectroscopy, flow cytometry, 16S qPCR, 16S qRT-PCR, ddPCR, and reference spike-in from various biological angles and provided a systematic decision-making scheme for future microbial investigations.

## Figures and Tables

**Table 1:** Absolute quantification methods in microbiome studies.

Absolute Quantification Method	Major Applications (Published)	Advantages	Limitations/Concerns	References
Fluorescence spectroscopy	Aquatic, soil, food and beverage, and air	High affinity; multiple dye selection to distinguish both live and dead cells	Fail to stain dead cells with complete DNA degradation; some dyes bind both DNA and RNA	Gordon et al., 2017, Guzaev et al., 2017, Saint-Ruf et al., 2010, Sieracki et al., 1999, Auty et al., 2001,
CARD-FISH + flow cytometry/qPCR	Aquatic	Direct quantification of specific taxa; detects both live and dead cells; provides insights for function, morphology, and ecology among taxa	Large population of cells are required for rare taxa detection; possibility of unspecific probe binding; Sample fixation may cause operation and efficiency biases; background noise	Hinzke et al., 2021, Kuo et al., 2021, Piwosz et al., 2021, Priest et al., 2021, Neuenschwander et al., 2015, Kubota et al., 2013
Flow cytometry	Feces, aquatic, and soil	Rapid; single cell enumeration; flexible parameters based on physiological characteristics; capability to differentiate live and dead cells	Background noise exclusion may be required; gating strategy; dilution may be required; not ideal for complex systems/heterogeneous samples	Luhung et al., 2021, Heinrichs et al., 2021, Xu et al., 2021, Zhu et al., 2019, Deng et a., 2019, Vandeputte et al., 2017, Prest et al., 2013, Berney et al., 2007, Longnecker et al., 2005, Salcher et al., 2011
Spike-in with internal reference	Soil, sludge, and feces	Rapid; easy incorporation into high throughput sequencing; high sensitivity; easy handling	Internal reference, spiking amount, and spiking time point can greatly affect the accuracy; 16S rRNA copy number calibration possibly needed.	Yang et al., 2018, Tourlousse et al., 2017, Smets et al., 2016, Lou et al., 2018, Stämmler et al., 2016
16S qPCR	Feces, clinical (lung), soil, plant, air, and aquatic	Directly quantifies specific taxa; cost-effective and easy handling; high sensitivity; compatible with low biomass samples	16S rRNA copy number calibration may be needed; PCR-related biases exist; standard curves are required	Luhung et al., 2021, Callegari et al., 2021, Blaud et al., 2021, Lei et al., 2021, Jian et al., 2020, Vandeputte et al., 2017, Stoddard et al., 2015, Sze et al., 2014, Brankatschk et al., 2012
16S qRT-PCR	Clinical (joint infection), food safety, feces, sludge, water remediation, and soil	High resolution and sensitivity; directly quantifies specific taxa; detects active cells; compatible with low biomass samples	More of an approximation for protein synthesis than overall cell count; unstable RNA/RNA degradation; 16S rRNA copy number calibration may be needed	Ma et al., 2018, Johnston and Behrens, 2020, Bui et al., 2012, Boyer and Combrisson, 2013, Kim et al., 2014, Stoddard et al., 2015, Matsuda et al., 2009
ddPCR	Clinical (lung, bloodstream infection), air, feces, and soil	Applicable to low concentrations of DNA; directly quantify specific taxa; high throughput capabilities, and no standard curve needed; compatible with low biomass samples	Dilutions are required for high concentrated template; may require a large number of replicates	Luhung et al., 2021, Ahn et al., 2020, Zeng et al., 2020, Sze et al., 2014, Kim et al., 2014, Ziegler et al., 2019, Gobert et al., 2018

## Data Availability

Not applicable.
